# Supplementary material for: Saccharide analysis of onion outer epidermal walls
Source: Biotechnol Biofuels. 2021 Mar 15;14:66. doi: 10.1186/s13068-021-01923-z (PMC7962260; doi:10.1186/s13068-021-01923-z)
Supplement: Supplementary file 1 — Additional file 1: Table S1. Parameters used for the fit of 13C CP spectrum in Fig. 3a. [file 13068_2021_1923_MOESM1_ESM.docx]

**Additional file 1. Table S1.** Parameters used for the fit of ^13^C CP spectrum in Figure 3a.

| **δ [ppm]** | **Assignment** | **Amplitude** | **Width [ppm]** | **Integral [%]** |
| --- | --- | --- | --- | --- |
| Cellulose | | | | |
| 105.8 | i1 | 12 771 | 2.8 | 7.8 |
| 105.2 | s1 | 3 266 | 3.0 | 2.2 |
| 89.0 | i4 | 3 329 | 3.3 | 2.4* |
| 84.5 | s4 | 8 336 | 3.5 | 6.3* |
| 75.5 | i3 and s3/5 | 22 105 | 4.4 | 21.1 |
| 73.0 | i2/5 and s2 | 26 779 | 3.5 | 20.4 |
| 66.0 | i6 | 2 500 | 3.0 | 1.6 |
| 62.0 | s6 | 6 900 | 3.0 | 4.5 |
| Other major peaks | | | | |
| 100.3 | GalA1 | 8 506 | 2.9 | 5.4 |
| 79.2 | GalA4 | 5 449 | 1.6 | 1.9 |
| 69.8 | GalA3 | 17 283 | 3.8 | 14.2 |
| 63.8 |  | 4 000 | 2 | 1.7 |
| 61.0 |  | 3 900 | 0.4 | 0.3 |
| Other minor peaks | | | | |
| 45.4 |  | 631 | 25.6 | 3.4 |
| 33.7 |  | 6 165 | 2.9 | 3.8 |
| 30.5 |  | 4 336 | 1.4 | 1.3 |
| 26.5 |  | 1 821 | 4.3 | 1.7 |

* highlights values used to calculate the interior-to-surface ratios for cellulose, which is later used in Method 2 for quantifying polysaccharide composition in **Additional files 3** and **4**.
