# Supplementary material for: Saccharide analysis of onion outer epidermal walls
Source: Biotechnol Biofuels. 2021 Mar 15;14:66. doi: 10.1186/s13068-021-01923-z (PMC7962260; doi:10.1186/s13068-021-01923-z)
Supplement: Supplementary file 2 — Additional file 2. Procedures for spectral deconvolution. [file 13068_2021_1923_MOESM2_ESM.docx]

**Additional file 2. Procedures for spectral deconvolution**

Here is described the methodology employed to obtain complex ^13^C NMR spectra deconvolution presented in **Figure 3**. It should be noted that the peak position, shape, width, amplitude can all affect the result; therefore, prior knowledge of the representative linewidth and chemical shifts are a necessity to achieve a satisfactory fit. The representative chemical shift information of cell wall polysaccharides can be accessed at the Complex Carbohydrate Magnetic Resonance Database (CCMRD) at [www.ccmrd.org](http://www.ccmrd.org) [1].

**Step-by-step building of the simulated spectra**

First, deconvolution of the basic Cross Polarization (CP) spectrum (**Figure 3a**) is performed to obtain references and information on the chemical shift, linewidth, and intensity of cellulose peaks. It is straightforward to get a reasonable fit of the CP spectrum because only a small number of components are present. The information is then used to guide the fit of cellulose peaks in the quantitative MultiCP spectrum. In Dmfit software, the CP fit is directly loaded in the MultiCP experimental spectrum. After baseline correction, a normalization factor of 1.1 is applied to cellulose to match intensity levels of i4 and s4 peaks. Resulting cellulose amplitudes are locked, alongside chemical shifts and widths.

For CP fit convergence, 5 other major peaks and 4 minor ones are mandatory. 3 out of the 5 supplementary major peaks correspond to the most abundant constituent of the onion cell wall, galacturonic acid. We note that this assignment of CP spectral lines to GalA and cellulose is in good agreement with past literature on major polysaccharides found in onion cell wall. Then, complete MultiCP fit (**Figure 3c**) is built up by adding components, one sugar after the other. Final parameters are given in **Additional file 3**.It began with arabinose and rhamnose, as they both display a well resolved carbon (respectively A1 and R6) that can be fitted without overlap of other unknown sugars at this this stage. Resulting amplitudes and widths from these A1 and R6 sites are applied to all other arabinose and rhamnose carbons. It must be noted that for A1, a visual fit was done (in other words, parameters for A1 were manually adjusted; the ‘compute’ command in DmFit was not used). It is followed with galactose (G6), for which data is obtained after local fit of the 0 to 50 ppm region, which includes its resolved peak around 18 ppm. Then, to fill up the 68 to 80 ppm region (chemical shift interval where spectral overlap is too great to distinguish any resolved peak), results of the 160 to 180 ppm local fit are used. The latter is considered a good starting point, as it requires only 3 lines, including 2 attributed to the components of interest, GalA and GlcA. Once again, amplitude and widths are applied to all other carbon of GalA and GlcA. At this stage, visual inspection clearly shows that overall line shape requires corrections, as several amplitudes of these sites are overestimated. Thankfully, it is noticed that major improvement of the fitted line shape can be obtained with adjustment of some originally CP-given spectral lines. (This may be justified, as local fit reference of C1 of other main constituent GalA/GlcA give anyway different widths of C3 and C4 compared to CP spectrum fit.) Furthermore, i1 cellulose peak width adjustment is operated, allowing the introduction of the last major sugar, galactose, through its C1. This way, minor contribution of xylose (at 104.5 ppm) is also taken in account (no other chemical shift area could have given fit parameters for this sugar).

Automatic fit computation, with only amplitude and widths of major components (about 20 parameters) allowed to vary, does not converge to a satisfactory result, even after an hour of calculations. Therefore, a manual fit is required. Parameters adjustments are generally made from low to high field to minimize extent of changes. After obtaining a satisfactory fit, fine tuning of C4 region is performed to discriminate the different cellulose polymorphs. With this model they are assumed to yield similar broad components as in CP spectrum. Without ^13^C labelling, considered cellulose conformers are interior chains, hydrophilic and hydrophobic surface chains, respectively referred by i, s^f^ and s^g^ in the literature [2].

**Estimate molecular fraction using deconvolution data**

From all deconvoluted spectral lines (including those classified as others in **Additional file 3**), we add all integrals for polysaccharides and lipid. Ratio of one to another yield 85% sugars to 15% lipid (**Additional file 4**). To obtain polysaccharide ratios from one to another, we mostly average selected integrals from peaks that are clearly resolved. Practically, this generally correspond to consider numerical integrals from the same peaks from which the fit has been initiated. Then, these averages are divided to the total amount of polysaccharides. Obtained ratios are used to establish charts presented in **Figure 3**. Formulas to obtain them are explicated below Table S2. Exception is made for cellulose. Indeed, to alleviate uncertainties, we propose 2 integration models. In the first one (presented in **Figure 3 and Figure S1**), all integrals from broad C4 peaks of cellulose are added, resulting in considering i4 and s4 as a representative amount of the total cellulose content in onion cell wall. This method yields a trustworthy cellulose content but does not allow us to discriminate interior from surface cellulose. In a second model, in order to try to get conformer specific information, we use resolved i6 peak as the reference and infer total cellulose amount by adding the hypothetical s6 contribution equaling i6 integral modulated by i to s ratio inferred from classic CP experiment.

Estimating the error is quite challenging. We expect around 10% error margin of each reported value based on the current signal-to-noise ratios. Additional error margin should be added to account the uncertainty due to the manual fitting of the spectrum. Therefore, we are confident on discriminating major components (GalA, cellulose) from minor ones (arabinose, rhamnose, xyloglucan), but acute proportions within each category could be subject to significant variation, especially coming from the broader areas of the spectrum.

**Additional Reference:**

[1] Kang, X.; Zhao, W.; Dickwella Widanage, M.C.; Kirui, A.; Ozdenvar, U.; Wang, T. “CCMRD: A Solid-State NMR Database for Complex Carbohydrates”, Journal of Biomolecular NMR 74, 239-250 (2020).

[2] Wang, T.; Yang, H.; Kubicki, J.D.; and Hong, M. “Cellulose Structural Polymorphism in Plant Primary Cell Walls Investigated by High-Field 2D Solid-State NMR spectroscopy and Density Functional Theory Calculations”, Biomacromolecules 17, 2210-2222 (2016).
