# Supplementary material for: Saccharide analysis of onion outer epidermal walls
Source: Biotechnol Biofuels. 2021 Mar 15;14:66. doi: 10.1186/s13068-021-01923-z (PMC7962260; doi:10.1186/s13068-021-01923-z)
Supplement: Supplementary file 3 — Additional file 3: Table S2. Parameters used for the fit of 13C MultiCP spectrum in Fig. 3b, c. The relatively well resolved resonances (underlined) are used for estimating polysaccharide composition. [file 13068_2021_1923_MOESM3_ESM.docx]

**Additional file 3. Table S2.** Parameters used for the fit of ^13^C MultiCP spectrum in Figure 3b, c. The relatively well resolved resonances (underlined) are used for estimating polysaccharide composition.

| **δ [ppm]** | **Carbon number** | **Amplitude** | **Width [ppm]** | **Integral [%]** | **Contribution**  **Before normalization** | **Normalized fraction,**  **Method 1** | **Normalized fraction,**  **Method 2** |
| --- | --- | --- | --- | --- | --- | --- | --- |
| **GalA/GlcA** | | | | | | | |
| Molecule 1 | | | | | | | |
| 176.0 | 6 | 38 944 | 1.2 | 4.5 | 6.3 **^a^** |  |  |
| 100.3 | 1 | 32 000 | 2.2 | 6.6 |  |  |  |
| 79.0 | 4 | 32 000 | 2.0 | 6.0 |  |  |  |
| 71.3 | 5 | 15 000 | 3.0 | 4.2 |  | 47% | 42% |
| 69.8 | 3 | 41 000 | 1.8 | 6.9 |  |  |  |
| 68.6 | 2 | 9 000 | 2.2 | 1.9 |  |  |  |
| 54.1 | Me | 5 000 | 1.2 | 0.6 |  |  |  |
| Molecule 2 | | | | | | | |
| 177.9 | 6 | 943 | 0.6 | 0.1 | 0.1 |  |  |
| 98.1 | 1 | 1 800 | 0.8 | 0.1 |  |  |  |
| 78.3 | 3 | 943 | 0.5 | 0.1 |  | 0.7% | 0.7% |
| 72.5 | 2/5 | 1 886 | 0.6 | 0.1 |  |  |  |
| . | 4 | . | . | . |  |  |  |
| **Rhamnose (Rha)** | | | | | | | |
| 96.4 | 1 | 4 000 | 0.9 | 0.4 | 0.4 **^b^** | 3.0% | 2.8% |
| 79.8 | 2 | 5 730 | 0.9 | 0.5 |  |  |  |
| 72.2 | 4 | 7 000 | 0.9 | 0.6 |  |  |  |
| 71.3 | 3 | 5 450 | 1.1 | 0.5 |  |  |  |
| 68.3 | 5 | 5 730 | 0.9 | 0.5 |  |  |  |
| 17.8 | 6 | 5 730 | 0.9 | 0.5 |  |  |  |
| **Cellulose (i/s)** | | | | | | | |
| 105.7 | i1 | 11 500 | 1.7 | 1.8 | Method 1:  3.7 **^c^**  Method 2:  5.2 **^d^** | 28% | 35% |
| 90.0 | i^e^4 | 1 000 | 2.0 | 0.2 **^c^** |  |  |  |
| 89.0 | i^a/b^4 | 3 000 | 3.3 | 0.9 **^c^** |  |  |  |
| 87.0 | i^c/d^4 | 4 400 | 3.0 | 1.2 **^c^** |  |  |  |
| 84.5 | s4 | 5 000 | 3 | 1.4 **^c^** |  |  |  |
| 75.5 | i3 + s3/5 | 20 000 | 4.4 | 8.2 |  |  |  |
| 73.1 | i2/5 + s2 | 25 000 | 3.5 | 8.2 |  |  |  |
| 64.9 | i6 | 13 500 | 1.2 | 1.5 **^d^** |  |  |  |
| 60.9 | s6 | 5 000 | 3.5 | 1.6 |  |  |  |
| **Galactose (Gal)** | | | | | | | |
| 105.5 | 1 | 9 900 | 1.0 | 0.9 | 1.3 **^e^** | 9.3% | 8.3% |
| 79.0 | 4 | 9 900 | 1.0 | 0.9 |  |  |  |
| 75.6 | 5 | 12 000 | 1.2 | 1.4 |  |  |  |
| 74.5 | 3 | 15 000 | 1.0 | 1.4 |  |  |  |
| 73.1 | 2 | 17 500 | 1.0 | 1.6 |  |  |  |
| 62.8 | 6 | 67 000 | 0.6 | 3.5 |  |  |  |
| **Arabinose (Ara)** | | | | | | | |
| 108.7 | 1 | 9 200 | 1.1 | 1.0 | 0.9 **^f^** |  |  |
| 83.5 | 4 | 7 300 | 1.2 | 0.8 |  |  |  |
| 82.6 | 2 | 7 300 | 1.2 | 0.8 |  | 6.4% | 5.8% |
| 78.1 | 3 | 4 500 | 1.0 | 0.4 |  |  |  |
| 67.6 | 5 | 6 000 | 1.0 | 0.6 |  |  |  |
| **Xylose in xyloglucan (Xyl)** | | | | | | | |
| 99.6 | 1 | 2 000 | 1.6 | 0.3 | 0.3 |  |  |
| 74.3 | 3 | 2 000 | 1.6 | 0.3 |  |  |  |
| 72.5 | 2 | 2 000 | 1.6 | 0.3 |  | 2.2% | 2.0% |
| 70.2 | 4 | 2 000 | 1.6 | 0.3 |  |  |  |
| 62.0 | 5 | 5 000 | 1.6 | 0.8 |  |  |  |
| **Glucose in xyloglucan (Glc)** | | | | | | | |
| 104.5 | 1 | 7 100 | 1.2 | 0.8 | 0.5 ^g^ | 4.0% | 3.6% |
| 85.2 | 4 | 5 000 | 1.6 | 0.3 |  |  |  |
| **Other signals of Polysaccharides** | | | | | | | |
| 174.4 |  | 2 817 | 0.8 | 0.2 |  |  |  |
| 60.9 |  | 220 000 | 0.4 | 8.2 |  |  |  |
| 21.6 | Ac^Me^ | 9 137 | 1.1 | 0.9 |  |  |  |
| **Lipid** | | | | | | | |
| 173.3 |  | 5 925 | 0.6 | 0.3 |  |  |  |
| 57.1 |  | 18 000 | 0.8 | 1.4 |  |  |  |
| 50.4 |  | 5 925 | 0.6 | 0.3 |  |  |  |
| 35.0 |  | 14 548 | 0.9 | 1.3 |  |  |  |
| 33.3 |  | 4 243 | 0.7 | 0.3 |  |  |  |
| 30.3 |  | 79 337 | 1.0 | 7.6 |  |  |  |
| 28.8 |  | 15 059 | 0.6 | 0.8 |  |  |  |
| 27.8 |  | 15 844 | 0.8 | 1.3 |  |  |  |
| 26.9 |  | 10 417 | 0.9 | 0.9 |  |  |  |
| 25.9 |  | 14 548 | 0.8 | 1.1 |  |  |  |
| 14.8 |  | 3 021 | 0.7 | 0.2 |  |  |  |

**^a^** The amount of molecule 1 in GalA/GlcA/Rha is calculated by averaging the resolved C1 and C4 peaks.

**^b^** The amount of Rha is calculated by averaging the resolved C1 and C6 (CH_3_) peaks.

**^c^** In method 1, the amount of cellulose is calculated by adding all the C4 resonances (interior and surface chains).

**^d^** In method 2, the amount of cellulose is calculated using the relatively resolved i6 peak, and the back calculate the amount of all cellulose using the interior-to-surface ratio obtained in CP spectrum.

**^e^** Galactose is component added in the last step, with high uncertainty. Therefore, intensities of all carbon sites are averaged.

**^f^** The amount of arabinose is calculated by averaging the C1, C2 and C4 peaks.

**^g^** The backbone of xyloglucan (XyG) is the average of the C1 and C4 peaks.
