# Supplementary material for: Saccharide analysis of onion outer epidermal walls
Source: Biotechnol Biofuels. 2021 Mar 15;14:66. doi: 10.1186/s13068-021-01923-z (PMC7962260; doi:10.1186/s13068-021-01923-z)
Supplement: Supplementary file 4 — Additional file 4: Table S3. Molecular composition derived from 13C MultiCP spectrum in Fig. 3b, c. The relatively well resolved resonances (underlined) are used for estimating polysaccharide composition. Carbohydrate peaks account for 85% of total intensity and lipid polymers account for 15% of all carbons. [file 13068_2021_1923_MOESM4_ESM.docx]

**Additional file 4. Table S3.** Molecular composition derived from ^13^C MultiCP spectrum in Figure 3b, c. The relatively well resolved resonances (underlined) are used for estimating polysaccharide composition. Carbohydrate peaks account for 85% of total intensity and lipid polymers account for 15% of all carbons.

|  | Carbohydrate | Lipid |
| --- | --- | --- |
| Fraction | 84.5% | 15.5% **^a^** |

|  | GalA/GlcA | Rha | Cellulose **^b^** | Gal | Ara | XyG **^c^** |
| --- | --- | --- | --- | --- | --- | --- |
| Method 1 | 47% | 3% | 28% | 9% | 6% | 6% |
| Method 2 | 42% | 3% | 35% | 8% | 5% | 6% |

**^a^** The carbohydrate resonances in **Additional file 3** account for 84.5% of all carbons. The lipid resonances account for 15.5 % of all carbons in the sample.

**^b^** In Method 1, the cellulose content is calculated by adding all the underlying carbon 4 resonances from 84 to 90 ppm. In Method 2, the cellulose content is calculated using only the signal from i6 peak in MultiCP, and the interior-to-surface ratio in CP.

**^c^** XyG amount is the sum of xylose and glucose in **Additional file 3**. Note that significant uncertainty is present with the amount of XyG as it is poorly resolved.
