# Supplementary material for: Saccharide analysis of onion outer epidermal walls
Source: Biotechnol Biofuels. 2021 Mar 15;14:66. doi: 10.1186/s13068-021-01923-z (PMC7962260; doi:10.1186/s13068-021-01923-z)
Supplement: Supplementary file 5 — Additional file 5: Figure S1. MultiCP integration models. [file 13068_2021_1923_MOESM5_ESM.docx]

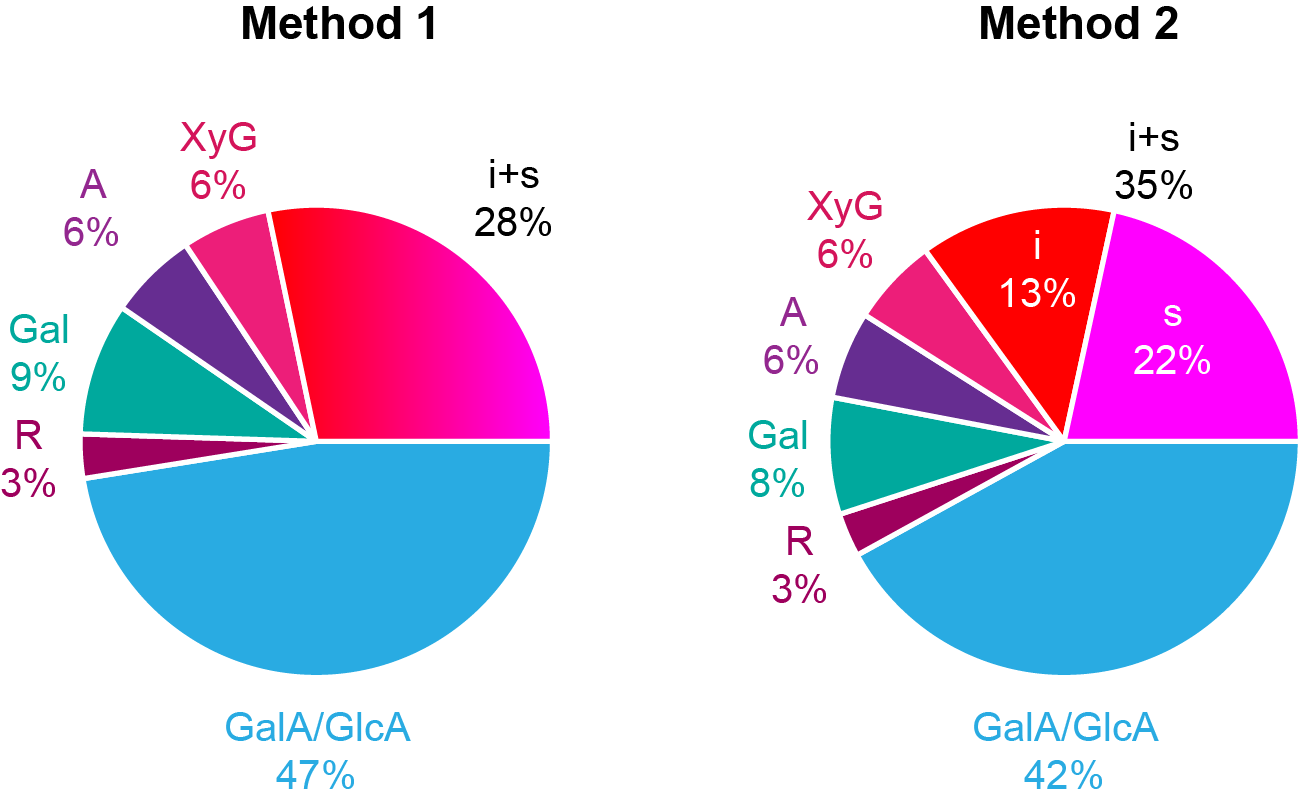


**Additional file 5. Figure S1.** MultiCP integration models. **Method 1:** For each sugar, except cellulose, percentages are obtained by averaging MultiCP integrals of resolved peaks (underlined values in **Additional file 3**, to obtain each sugar contribution), and dividing the result by the sum of all contributions (including cellulose one). For cellulose, as C4 region of MultiCP is broad and poorly defined, i and s contributions come from the sum of all peaks in this region. **Method 2:** Sugar contributions are obtained with the same method. However, for cellulose, this time resolved s6 peaks on MultiCP is used as a reference. i and s contributions are obtained by multiplying this integral by i to s ratio obtained from the usual ratio of C4 integrals in the classic CP experiment. We note that this second method clearly over-estimate total cellulose content as i6 MultiCP deconvolution component is particularly difficult to obtain. i (interior cellulose), s (surface cellulose), Gal (galactose), A (arabinose), R (rhamnose), and XyG (xyloglucan).
